# A simulation study of sample size for DNA barcoding

**DOI:** 10.1002/ece3.1846

**Published:** 2015-12-01

**Authors:** Arong Luo, Haiqiang Lan, Cheng Ling, Aibing Zhang, Lei Shi, Simon Y. W. Ho, Chaodong Zhu

**Affiliations:** ^1^ Key Laboratory of Zoological Systematics and EvolutionInstitute of ZoologyChinese Academy of SciencesBeijing100101China; ^2^ School of Statistics and MathematicsYunnan University of Finance and EconomicsKunming650221China; ^3^ Department of Computer Science and TechnologyCollege of Information Science and TechnologyBeijing University of Chemical TechnologyBeijing100029China; ^4^ College of Life SciencesCapital Normal UniversityBeijing100048China; ^5^ School of Biological SciencesUniversity of SydneySydneyNew South Wales2006Australia; ^6^ College of Life SciencesUniversity of Chinese Academy of SciencesBeijing100049China

**Keywords:** Coalescence, haplotype, maximum pairwise distance, mismatch distribution, nucleotide diversity

## Abstract

For some groups of organisms, DNA barcoding can provide a useful tool in taxonomy, evolutionary biology, and biodiversity assessment. However, the efficacy of DNA barcoding depends on the degree of sampling per species, because a large enough sample size is needed to provide a reliable estimate of genetic polymorphism and for delimiting species. We used a simulation approach to examine the effects of sample size on four estimators of genetic polymorphism related to DNA barcoding: mismatch distribution, nucleotide diversity, the number of haplotypes, and maximum pairwise distance. Our results showed that mismatch distributions derived from subsamples of ≥20 individuals usually bore a close resemblance to that of the full dataset. Estimates of nucleotide diversity from subsamples of ≥20 individuals tended to be bell‐shaped around that of the full dataset, whereas estimates from smaller subsamples were not. As expected, greater sampling generally led to an increase in the number of haplotypes. We also found that subsamples of ≥20 individuals allowed a good estimate of the maximum pairwise distance of the full dataset, while smaller ones were associated with a high probability of underestimation. Overall, our study confirms the expectation that larger samples are beneficial for the efficacy of DNA barcoding and suggests that a minimum sample size of 20 individuals is needed in practice for each population.

## Introduction

Over the past decade, DNA barcoding has proven to be a useful tool in studies of taxonomy, ecology, biodiversity assessment, and various other fields (Waugh 2007; Valentini et al. 2009; Scheffers et al. 2012). And its concept has become the basis of DNA mini‐barcoding (Meusnier et al. 2008) and DNA metabarcoding which uses high‐thoughput sequences from environmental samples (Yu et al. 2012). Nevertheless, many theoretical and methodological aspects of DNA barcoding remain subject to debate, including the species concepts (Rubinoff et al. 2006a,b), variability in the success of the method (e.g. Meier et al. 2006; Dasmahapatra et al. 2010), and the choice of molecular markers (Roe and Sperling 2007; Luo et al. 2011). In particular, the impact of sample size has long been an important issue in DNA barcoding (Austerlitz et al. 2009; Zhang et al. 2010; Bergsten et al. 2012; Jin et al. 2012).

Although DNA barcoding aims to offer a rapid, reliable, automatic, and cost‐effective method for species identification and delimitation, it can be complicated by variation in levels of genetic polymorphism among species (Hebert et al. 2003; Austerlitz et al. 2009). The accuracy and efficacy of DNA barcoding generally depend on the existence of a gap between intraspecific variation and interspecific variation, but this gap is absent when species are polyphyletic or paraphyletic (Meyer and Paulay 2005; Austerlitz et al. 2009). This criterion is becoming less important with the advent of methods that do not entirely rely on pairwise genetic distances, including those that employ an explicit phylogenetic framework (e.g. Pons et al. 2006). In any case, a detailed understanding of intraspecific polymorphism in different species forms the basis of reliable DNA barcoding via both traditional and new methods, and is particularly important for constructing reference databases. In turn, this is highly dependent on the degree of sampling per species. In practice, however, there is usually a compromise between the degree of sampling per species and the extent of taxonomic coverage, given limited resources for conducting genetic sampling. A consequence is that intraspecific sampling is often quite limited (Meyer and Paulay 2005; Zhang et al. 2010; Bergsten et al. 2012; Liu et al. 2012).

There have been a number of studies into the impacts of sample size on DNA barcoding. Among these, Matz and Nielsen (2005) found that at least 12 individuals per species were needed to achieve confidence in their statistical method for testing species membership, while 5 and 12 references per species at least were respectively proposed by others (Ross et al. 2008; Jin et al. 2012). When comparing phylogenetic and statistical classification methods for DNA barcoding, Austerlitz et al. (2009) found that the success rate increased with sample size. Zhang et al. (2010) examined the increase in haplotype richness with sample size based on a nonparametric resampling approach. In a study of beetles, Bergsten et al. (2012) showed that a large sample size (~70 individuals) was required to obtain a reliable estimate of 95% of the intraspecific variation of *Agabus bipustulatus* (Insecta: Coleoptera: Dytiscidae) throughout Europe. Conversely, a plant barcoding study of *Taxus* species (Pinopsida: Pinales: Taxaceae) suggested that sampling a single individual per population was adequate (Liu et al. 2012). Each of these studies focused on a specific method, a particular aspect of genetic polymorphism, or a specific taxon. This points to a need for a more comprehensive analysis of the performance of various estimators of genetic polymorphism when there is limited sampling.

Here, we analyse the impact of sample size on DNA barcoding. Using DNA sequence data generated via simulation under a coalescent model, we examined the behaviour of four estimators of genetic polymorphism: mismatch distribution, nucleotide diversity, the number of haplotypes, and maximum pairwise distance.

## Materials and Methods

### Coalescent assumptions

The coalescent framework captures ancestor‐descendant relationships under the Wright‐Fisher model (Fisher 1922; Wright 1931), and has been widely used to study the evolutionary process at the population level (Kingman 1982). Simple coalescent models typically include assumptions of a haploid genealogy, absence of recombination, absence of natural selection, and a constant mutation rate. These are consistent with most animal DNA barcoding studies, which widely employ the mitochondrial barcode cytochrome c oxidase 1 (*CO1*). In addition, many studies focus on the biodiversity of particular geographic regions; here we examine a simple scenario involving a single population or deme.

### Data simulation

The program *makesamples* (*ms*) was used to simulate DNA evolution based on the coalescent (Hudson 2002). We generated random genealogies, on which mutations were randomly added according to a Poisson distribution with a constant mutation rate. We assumed *θ = *4*Nμ = *3.0 for each population, where *θ* is the mutation parameter, *N* is the population size, and *μ* is the mutation rate. We drew 500 samples for each of 10 independent replicates. A constant population size was assumed in these simulations. We considered more complex population‐size histories, but have not included them in the present study (see Discussion for details).

With the genealogies simulated by *ms* (Fig. S1), we used *Seq‐Gen 1.3.2* (Rambaut and Grassly 1997) to simulate the evolution of nucleotide sequences under finite‐sites models. We used the Jukes‐Cantor (JC) model of nucleotide substitution (Jukes and Cantor 1969). To approximate the length of the mitochondrial *CO1* gene, simulated sequences had lengths of 1,500 bp. We rescaled the branch lengths to make them equal to the expected number of substitutions per site. This was done on a case‐by‐case basis so that intraspecific genetic variance was always less than 3%.

**Figure 1:**
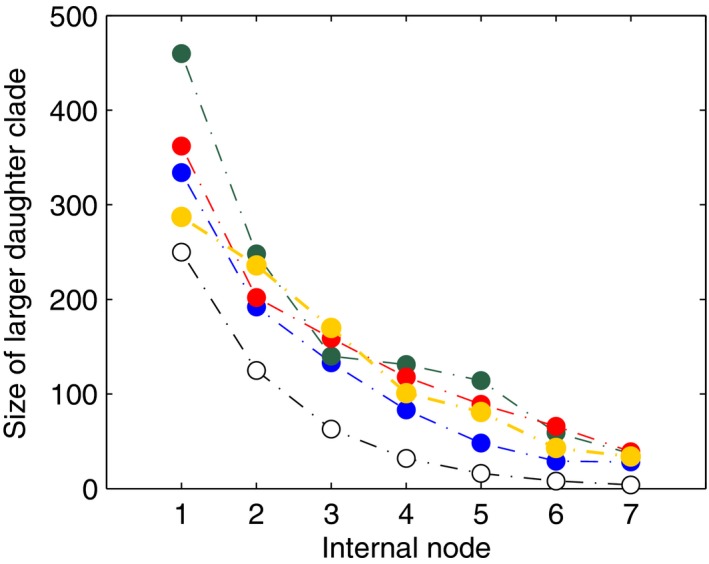
Split information around internal nodes of four chosen genealogies. The *x*‐axis represents seven internal nodes beginning at the root, while the *y*‐axis represents the size of the larger daughter clade. Among the ten trees consisting of 500 tips, data are shown here for tree_A (blue solid circles), tree_B (green solid circles), tree_F (red solid circles), and tree_I (yellow solid circles). Empty black circles represent data from a balanced tree topology.

To evaluate the tree shape of the simulated genealogies, we calculated the *Colless* index (Colless 1982; Heard 1992), using the *R* package *apTreeshape* (Bortolussi et al. 2005), yielding a small range of values (0.0173 to 0.0247). In addition, following the method of Aldous (2001), we plotted split information of the internal nodes near the root to give evidence of different branching patterns (Fig. 1).

### Mismatch distribution

Pairwise sequence comparisons are important for estimating intraspecific and/or interspecific genetic variances for DNA barcoding. This approach is commonly used in the form of mismatch distributions, which plot the frequency distribution of pairwise distances from a population sample. The distribution is multimodal for populations of constant or rapidly shrinking size and unimodal for populations that have experienced rapid growth (Slatkin and Hudson 1991; Rogers and Harpending 1992; Schenekar and Weiss 2011). We examined the influence of sample size on mismatch distributions using our 10 simulated datasets. For each dataset, we computed pairwise JC distances using PAUP* v4.0b10 (Swofford 2002). To allow straightforward comparisons among datasets, we standardized the pairwise distances in each dataset using min‐max normalization (e.g. Jain et al. 2005). For example, for dataset seq_A:

(1) $$d_{i}^{\prime }=\frac{d_{i}-{\text{min}}_{A}}{{\text{max}}_{A}-{\text{min}}_{A}}\times \left(\text{new}\_{\text{max}}_{A}-\text{new}\_{\text{min}}_{A}\right)+\text{new}\_{\text{min}}_{A}$$

where $d_{i}^{\prime }$ is the corrected distance corresponding to the original distance *d*_*i*_, min_*A*_ and max_*A*_ are the respective minimum and maximum pairwise distances from seq_A, new_max_*A*_ is 0.03, and new_min_*A*_ is 0.00. We then plotted histograms together with kernel density estimates (Silverman 1981) and a heatmap to show the distribution of pairwise distances. For each dataset of 500 sequences, we drew random subsamples of 5, 10, 20, 30, 50, and 100 sequences. Subsampling was done 10 times for each sample size. Their mismatch distributions were then compared with that of the full dataset. With pairwise distances normalized in the full dataset, further standardization was not needed for these subsamples.

### Nucleotide diversity

Nucleotide diversity (*π*), the average of all pairwise distances in a sample (Nei and Li 1979; Nei and Miller 1990), is commonly used to estimate genetic polymorphism and forms the basis of further tests (e.g. Tajima's D test; Tajima 1989). In the context of DNA barcoding, *π* can be treated as the mean of the pairwise distances within species of interest (e.g. Luo et al. 2011; Porco et al. 2014). It can be calculated as

(2) $$\pi =\frac{X_{1}+X_{2}+X_{3}+\cdots +X_{k}}{k}$$

where *X* is the pairwise distance and *k* is the number of pairwise comparisons in a sample of size *n*. A sample size (*n*) of at least nine provides more than 30 pairwise comparisons (*k*), thus forming one statistically large sample. According to the Lindeberg‐Lévy central limit theorem, if *k* is large enough, the distribution of *π* tends to follow a normal distribution with mean equal to *β* and variance equal to *σ* ^2^/*k*, where *β* and *σ* are the nucleotide diversity and variance of pairwise distances of the full dataset respectively. That is,

(3) $$\sqrt{k}{\left((\frac{1}{k}\sum_{i=1}^{k}X_{i})-\beta \right)}^{d}\to N\left(0,\sigma^{2}\right).$$

This holds regardless of whether pairwise distances of the entire dataset fall into a bell‐shaped distribution or not. We investigated this using the simulated data. We drew subsamples of 2, 5, 10, 20, 30, 40, 50, 60, 70, 80, 90, and 100 sequences with 10,000 replicates for each sample size. For each subsample, we computed nucleotide diversity based on pairwise JC distances.

### Number of haplotypes

The number of different haplotypes is an important indicator of genetic diversity in studies of populations. We used our simulated data to examine the effect of sample size on the number of haplotypes. We drew subsamples of 2, 10, 20, 30, 40, 50, 60, 70, 80, 90, 100, 110, 120, 130, 140, and 150 sequences with 100 replicates for each sample size. For each subsample, the number of haplotypes was computed using the software *DnaSP v5.10.01* (Rozas and Rozas 1995; Librado and Rozas 2009). The Michaelis‐Menten equation was to analyse the median number of haplotypes from each resultant set of 100 replicates (Zhang et al. 2010):

(4) $$F\left(x\right)=\frac{ax}{1+bx}$$

where *F*(*x*) represents the median number of haplotypes and is the function of the sample size, *x*, and constants *a* and *b* were computed by nonlinear fitting via least‐squares estimation (Tang 2008) across the 16 different subsample sizes.

### Maximum pairwise distance

Maximum pairwise distance is a simple representation of the genetic diversity in a sample. If molecular evolution has been clocklike, correctly identifying the maximum genetic distance is equivalent to capturing the most recent common ancestor of all present‐day individuals in the gene tree. This can require a relatively large number of samples, especially if the gene tree is imbalanced (Sanderson 1996). We tested the effect of sample size on this measure, with reference to the maximum pairwise distance of the full dataset. Subsamples of sizes 2, 5, 10, 20, 30, 40, 50, 60, 70, 80, 90, and 100 were drawn from each dataset of 500 sequences, with 10,000 replicates for each sample size. We then compared the maximum pairwise JC distance of subsamples to that of corresponding full dataset.

### Additional datasets

We repeated all of our analyses using two additional datasets, containing 300 sequences (dataset seq_K) and 1000 sequences (dataset seq_L) respectively. These datasets were generated using the same procedure as for the 500‐sequence datasets described above. We analysed mismatch distributions using random subsamples of 5, 10, 20, 30, 50, and 100 sequences, with 10 replicates for each sample size. We estimated nucleotide diversity and maximum pairwise distance using random subsamples of 2, 5, 10, 20, 30, 40, 50, and 60 sequences, with 10,000 replicates for each sample size. We calculated the number of haplotypes from random subsamples of 2, 20, 40, 60, 80, 100, 120, and 140 sequences, with 100 replicates for each sample size.

## Results

### Effect of sample size on mismatch distribution

**Figure 2:**
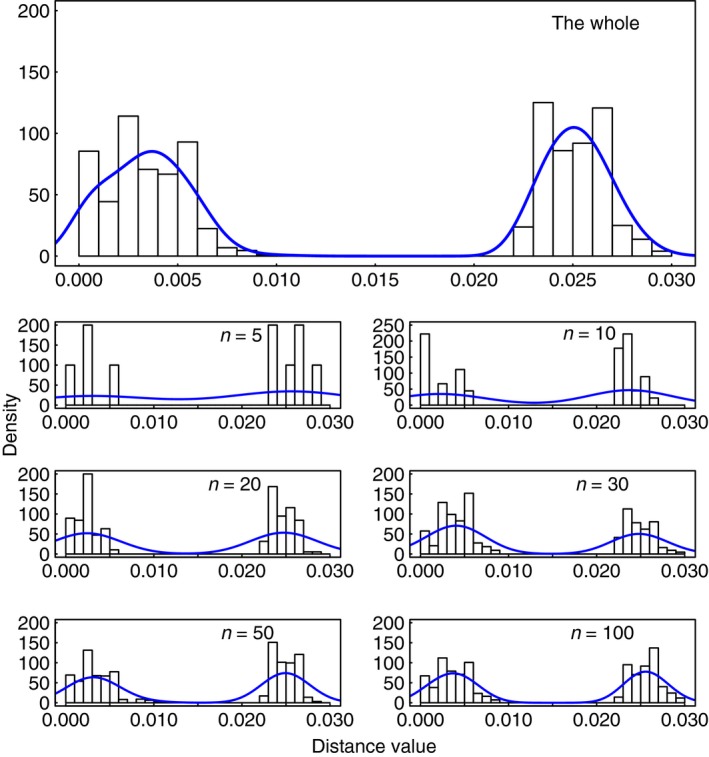
Mismatch distributions together with kernel density estimates of dataset seq_I and its subsamples. Only the result from one randomly chosen subsample of each size is shown here.

The mismatch distributions of the 10 full datasets were distinct from each other in shape, although all were bimodal or multimodal (Fig. 2; Data S1). For some datasets (e.g. seq_I in Fig. 2), there were gaps in the distribution of pairwise distances, as we normalized the pairwise distances within each dataset. The heatmap and related clustering indirectly show 124,750 values for each dataset and the relationships among the 10 datasets (Fig. S2).

To characterize the impact of sample size on mismatch distributions, we focused on three features: the range of distance values, existence of large gaps, and approximate position of the modes. The mismatch distributions for large sample sizes (50 and 100) bore a close resemblance to that of the corresponding full dataset. In contrast, when the sample size was only 5 or 10, the mismatch distributions bore little resemblance to those of the full datasets; the distributions from the subsamples contained additional gaps and the curves of kernel‐density estimates had uncertain shapes. At intermediate sample sizes, the shapes of the mismatch distributions were variable among replicates but were broadly similar to those of the full dataset.

### Effect of sample size on nucleotide diversity

**Figure 3:**
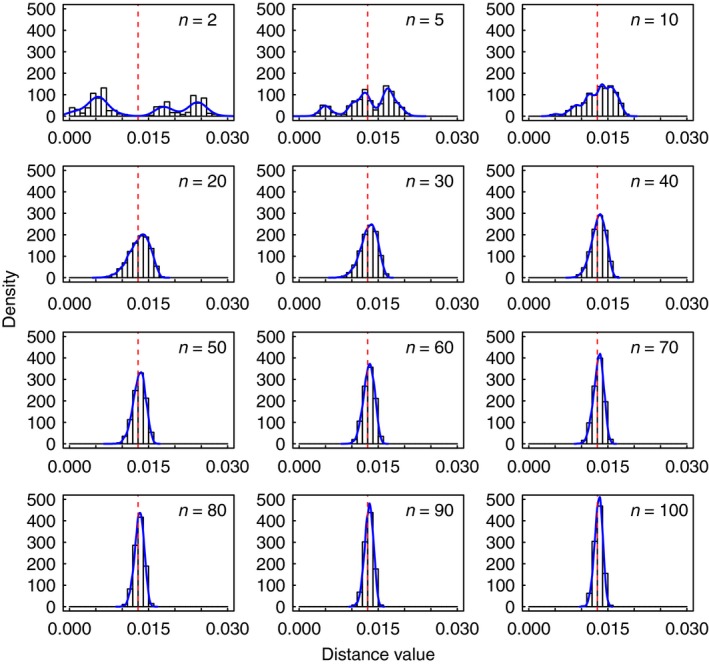
Histograms showing distributions of nucleotide diversity values of subsamples from dataset seq_J. The blue curves are from kernel density estimates, while the red vertical lines indicate nucleotide diversity of the full dataset.

The distributions of nucleotide diversity from random subsamples of the data (with size ranging from 2 to 100) shared a number of features, verifying the central limit theorem to some degree (Fig. 3; Data S2). When the sample size was 2 or 5, the distributions of the 10,000 computed values of nucleotide diversity (*π*) were usually not bell‐shaped, with a number of values distinct from the nucleotide diversity of the full dataset (*β*, denoted by the red vertical line in Fig. 3 and Data S2). When the sample size was 10, the distributions generally approached the bell curve but with some variation among datasets. When the sample sizes were 20 and greater, the distributions of nucleotide diversity were generally bell‐shaped; among subsamples from the same dataset, the mode of every set of 10,000 values closely approximated the nucleotide diversity of the full dataset; the number of outlier values declined with increasing sample size.

To examine the distributions in greater detail, we calculated the percentage of values in range of *β *± 0.001. We found that the larger the size of the random sample, the more closely its nucleotide diversity approached the expected value (Table 1). Although 100% of values fell within the target range when the sample size was 100, the percentage dropped to 87.80% when the sample size was only 10 for seq_A. With sampling from the 10 datasets (i.e. from seq_A to seq_J in Table 1) with a size of 20, percentages ranged from 52.19% to 92.18%. This suggests that descriptive statistics might not present a full picture of the findings. In Table 1, the mean of 10,000 nucleotide diversity values was very similar to *β*, whether the sample size was 2 or 100.

**Table 1:** Descriptive statistics of nucleotide diversities. Each of the ten datasets (from seq_A to seq_J) contains 500 simulated sequences, while seq_K and seq_J contain 300 and 1000 sequences, respectively

Dataset	*β*	Mean value of *π*s											
		Percent of values in range of *β *± 0.001											
		2^a^	5^a^	10^a^	20^a^	30^a^	40^a^	50^a^	60^a^	70^a^	80^a^	90^a^	100^a^
Seq_A	0.014	0.014	0.014	0.014	0.014	0.014	0.014	0.014	0.014	0.014	0.014	0.014	0.014
		0.00%	36.73%	87.80%	61.97%	89.11%	94.99%	97.27%	98.17%	98.61%	99.37%	99.62%	99.75%
Seq_B	0.012	0.012	0.012	0.012	0.012	0.012	0.012	0.012	0.012	0.012	0.012	0.012	0.012
		8.17%	32.13%	46.32%	71.65%	83.74%	89.64%	93.58%	95.84%	97.44%	98.10%	98.78%	99.05%
Seq_C	0.011	0.011	0.011	0.011	0.011	0.011	0.011	0.011	0.011	0.011	0.011	0.011	0.011
		0.64%	27.28%	37.39%	55.44%	65.86%	74.53%	79.13%	82.52%	85.99%	88.96%	90.57%	91.98%
Seq_D	0.009	0.009	0.009	0.009	0.009	0.009	0.009	0.009	0.009	0.009	0.009	0.009	0.009
		4.40%	58.52%	81.59%	92.18%	96.90%	98.70%	99.46%	99.81%	99.96%	99.96%	100.00%	100.00%
Seq_E	0.012	0.012	0.012	0.012	0.012	0.012	0.012	0.012	0.012	0.012	0.012	0.012	0.012
		29.19%	40.57%	61.32%	80.06%	88.82%	93.06%	96.20%	97.77%	98.94%	99.20%	99.48%	99.76%
Seq_F	0.010	0.010	0.010	0.010	0.010	0.010	0.010	0.010	0.010	0.010	0.010	0.010	0.010
		19.79%	50.49%	75.99%	90.58%	96.14%	97.71%	98.68%	99.29%	99.67%	99.85%	99.89%	99.94%
Seq_G	0.012	0.012	0.012	0.012	0.012	0.012	0.012	0.012	0.012	0.012	0.012	0.012	0.012
		6.23%	35.48%	56.93%	80.71%	90.35%	95.30%	97.96%	99.06%	99.45%	99.76%	99.92%	99.92%
Seq_H	0.005	0.005	0.005	0.005	0.005	0.005	0.005	0.005	0.005	0.005	0.005	0.005	0.005
		20.19%	8.03%	32.76%	60.49%	72.78%	77.77%	83.80%	88.68%	92.31%	93.81%	95.75%	96.62%
Seq_I	0.013	0.013	0.013	0.013	0.013	0.013	0.013	0.013	0.013	0.013	0.013	0.013	0.013
		0.00%	30.79%	78.59%	90.98%	94.87%	97.22%	98.70%	98.95%	99.41%	99.59%	99.71%	99.85%
Seq_J	0.013	0.013	0.013	0.013	0.013	0.013	0.013	0.013	0.013	0.013	0.013	0.013	0.013
		0.05%	26.03%	35.84%	52.19%	63.23%	71.47%	77.83%	83.00%	86.59%	89.20%	91.91%	93.84%
Seq_K	0.008	0.008	0.008	0.008	0.008	0.008	0.008	0.008	0.008	NA	NA	NA	NA
		4.77%	56.27%	78.72%	87.34%	91.99%	94.79%	96.85%	98.01%	NA	NA	NA	NA
Seq_L	0.010	0.010	0.010	0.010	0.010	0.010	0.010	0.010	0.010	NA	NA	NA	NA
		22.75%	24.00%	43.79%	64.91%	75.38%	82.92%	87.62%	90.91%	NA	NA	NA	NA

a Size of subsamples that were drawn randomly from the full dataset.

### Effect of sample size on the number of haplotypes

**Figure 4:**
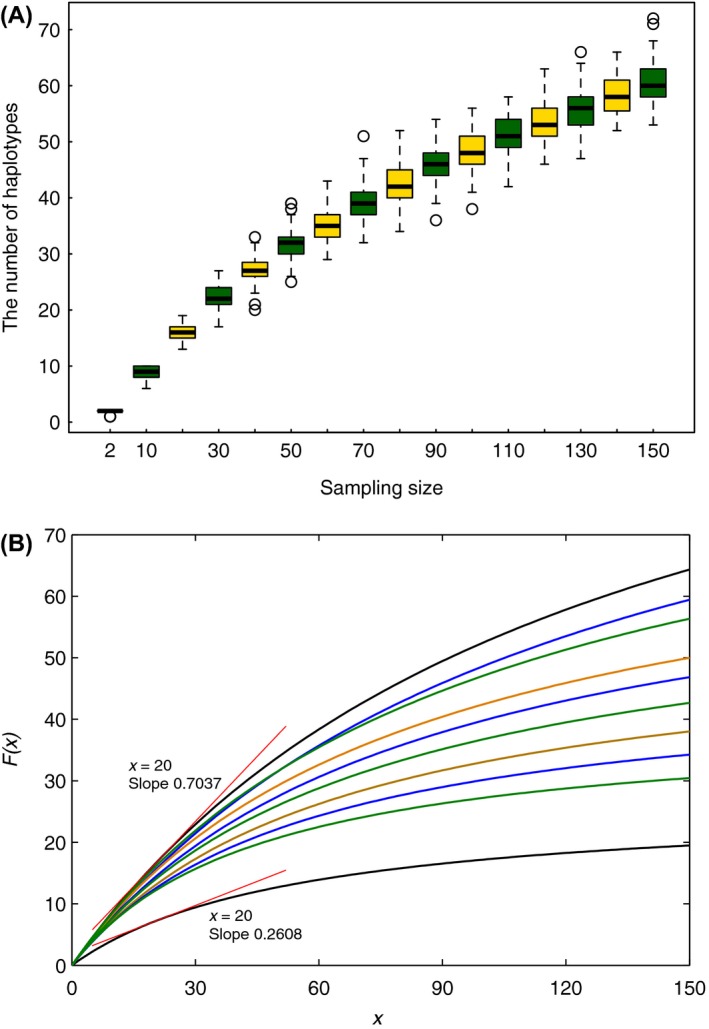
(A) Boxplots showing the numbers of haplotypes for every 100 repeats of subsamples of the same size from dataset seq_C. The *x*‐axis denotes the sample size, while the *y*‐axis represents the detailed number of haplotypes. (B) Ten asymptotic‐logarithm curves corresponding to the ten Michaelis‐Menten equations, which were estimated from the median values in boxplots of datasets from seq_A to seq_J.

Larger samples yielded greater numbers of haplotypes, but with generally larger deviations from the medians despite the fact that there was a declining growth in deviations with increasing sample sizes (Fig. 4A; Data S3). With any quartile as the reference, there tended to be fewer newly added haplotypes as the sample size increased.

Our estimates of the constants *a* and *b* in the Michaelis‐Menten equation showed that the variance of the error term ranged from 0.1524 to 0.5042. Although functions representing the ten Michaelis‐Menten equations all yielded asymptotic‐logarithm curves (Fig. 4B), the same sample size could lead to different numbers of haplotypes, especially for large sample sizes. Given the same sample size (e.g. 20), the slopes at the corresponding points in the curves were different (e.g. 0.2608 and 0.7037; Fig. 4B), reflecting the fact that different sample sizes would be required for the 10 curves with slope values of zero.

### Effect of sample size on maximum pairwise distance

**Figure 5:**
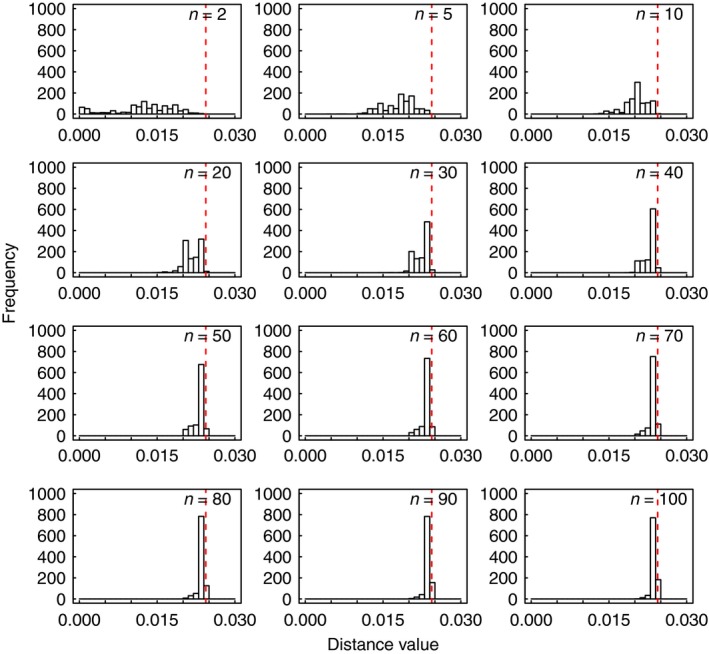
Histograms showing distributions of maximum pairwise distances of subsamples from dataset seq_E. The red vertical lines indicate maximum pairwise distance of the full dataset.

When sample sizes were 20 and greater, the maximum pairwise distance of the sample closely represented that of the full dataset (Fig. 5; Data S4). In contrast, when sample sizes were smaller (especially when the size was 2 or 5), the maximum pairwise distance of the sample tended to underestimate that of the full dataset, and values varied considerably among different samples.

### Results of additional datasets

Overall, the results from additional datasets were generally consistent with those described above for the 10 datasets containing 500 sequences each (Table 1; Data S1–S4). This confirmed that the effects of sample size did not depend on the size of the full simulated datasets.

## Discussion

Our results confirm the benefits of increasing sample sizes for four different measures of genetic polymorphism that are closely associated with DNA barcoding. Our findings are based on a simulation approach, which has several key benefits. First, with the assumption of random mating, the sequences in each dataset can be directly regarded as samples from the same geographic population or deme. This is in accordance with most DNA barcoding studies, which tend to focus on the biodiversity of particular geographic regions (Bergsten et al. 2012). Second, all of the simulated datasets were independent replicates, which can result in relatively generalized conclusion. Third, with our results being consistent across simulated samples of different sizes (i.e. 300, 500, and 1,000), we can conclude that our results can reasonably apply to the entire population. It is noteworthy that we assumed a constant population size for all simulations. Shrinking populations were not included, considering that there are usually limited individuals for sampling and may be no coalescence in their evolutionary history. Samples from exponentially growing populations should yield gene trees with longer external branches and their pairwise distances are expected to form a unimodal distribution (Slatkin and Hudson 1991; Schenekar and Weiss 2011). Given that this is a simpler case than that of constant‐size populations, we chose not to include growing populations in the simulations performed in this study.

### The impact of sample size

Investigations of both simulated and real data have proposed that sample sizes should be maximized for each species, because this provides a more comprehensive picture of haplotype diversity (Zhang et al. 2010). As expected, our analysis of the number of haplotypes generally supports this recommendation (Fig. 4). Our study also considered the effect of sample size on mismatch distribution, nucleotide diversity, and maximum pairwise distance. Generally, the mismatch distributions of subsamples could be classified into two different groups: one of smaller sample sizes (i.e. 5 and 10) and the other of larger sample sizes (i.e. not fewer than 20). Unsurprisingly, larger samples produced distributions that bore closer resemblance to that of the full dataset. In the case of nucleotide diversity, with the exception of the smaller sample sizes (i.e. 2, 5, and 10), 10,000 repeats tended to yield distributions that were bell‐shaped. In addition, the larger the size of the random sample, the more closely its nucleotide diversity approached the expected value (Fig. 3; Table 1). The results from our investigation of maximum pairwise distance are consistent with the above in that the genetic diversity of the full dataset can be accurately estimated when the sample size were 20 and greater (Fig. 5). Thus, our study has confirmed that it is better to obtain samples as large as possible, with a minimum sample size of 20 individuals per population. When sampling from multiple populations, a case that was not addressed in the present study, stratified sampling would involve repeated sampling in all strata if an absence of gene flow can be assumed.

### Estimators of genetic polymorphism

In view of the entire population and its random sample, our study of the impact of sample size on DNA barcoding offers insights into the performance of several estimators of genetic polymorphism. Among these, the mismatch distribution can provide detailed depictions of the pairwise distances in a sample. With its other applications in demographic analysis, it is useful for constructing reference databases for DNA barcoding, providing information such as the approximate range of intraspecific distances and possible gaps existing in intraspecific distances. However, it is not so straightforward to use mismatch distributions to summarise genetic polymorphism, which is more complex in nature. For this purpose, the number of haplotypes is commonly employed instead. However, the number itself cannot present detailed information about the sequence data, which are important for delimiting species, estimating demographic parameters, and other evolutionary analyses. Moreover, although the number of haplotypes in the entire population can be inferred using the Michaelis‐Menten equation, such an approach is not practical for studies of real species. We found that the nucleotide diversity of a large sample can provide a good reflection of the genetic polymorphism of the entire population of interest. However, based on mismatch distributions where there are possible gaps and which are usually nonwave‐like, it is not always a good estimator for evaluating the central tendency of pairwise distances. Finally, we found that the maximum pairwise distance of a large sample provides a simple and straightforward means of summarizing the genetic diversity of the entire population.

### Other implications for DNA barcoding

Our results showed that there may be gaps in mismatch distributions of pairwise distances, even when the sample size was as large as 500. The existence of gaps is consistent with basic coalescent theory. Towards the root of the genealogy, there are fewer lineages and the branches tend to be longer; a greater number of mutations can accumulate along these basal branches, leading to a gap between the intra‐ and intergroup distances. In specific cases, the gap can be obscured stochastically, due to mutation rate variation over time or other factors. However, the possible existence of gaps in intraspecific distances does propose a potential problem for DNA barcoding with methods based on distances or gaps (e.g. Hebert et al. 2004; Puillandre et al. 2012), especially when the reference database has a limited number of sample sequences. Comparatively, Bayesian phylogenetic inference, the generalized mixed Yule‐coalescent method (Pons et al. 2006), and the Bayesian modeling approach (Yang and Rannala 2010) should be more reliable for DNA barcoding.

In practice, there is an increasing tendency to employ multiple genes for species delimitation (e.g. Yang and Rannala 2010; Dupuis et al. 2012; Satler et al. 2013). Our independent simulated data can be regarded as samples from different, unlinked loci of the same population. With the generally consistent results from these datasets, our findings on the impacts of sample size should also be applicable to multilocus DNA barcoding.

On the whole, our investigation of four estimators of genetic polymorphism confirms the benefit of increasing sample size. More importantly, we found that a sample size of 20 is able to provide a reasonable reflection of the polymorphism of the entire population. Yet, due to the basic assumptions involved in our approach, our results are only applicable for studies limited to a single geographic population. Our results also reveal some of the disadvantages of these estimators in evaluating genetic polymorphism. Other findings, such as the existence of gaps in mismatch distributions, have potential consequences for DNA barcoding and related studies. Compared with previous studies of sample sizes for DNA barcoding, our study presents a more systematic and comprehensive evaluation. Further work should aim to investigate more complex simulation conditions and provide empirical verifications.

## Conflict of Interest

None declared.

## Data Accessibility

Data files are currently available on request from the authors.

## Supporting information


**Figure S1.** Ten genealogies shown by rectangular phylogram. Since the branch lengths were rescaled while sequences being simulated, scale bars are not shown here.

**Figure S2.** Heatmap showing pairwise distances of the ten datasets (from seq_A to seq_J) together with hierarchical clustering.

**Data S1.** Mismatch distributions of all the datasets except seq_I. Kernel density estimates are provided except for dataset seq_H because of the data incompatibility with the estimate.

**Data S2.** Histograms showing distributions of nucleotide diversity values from all the datasets except seq_J.

**Data S3.** Boxplots showing the numbers of haplotypes from all the datasets except seq_C.

**Data S4.** Histograms showing distributions of maximum pairwise distances from all the datasets except seq_E.
