# Supplementary figures and images for: A simulation study of sample size for DNA barcoding
Source: Ecol Evol. 2015 Dec 1;5(24):5869–79. doi: 10.1002/ece3.1846 (PMC4717336; doi:10.1002/ece3.1846)

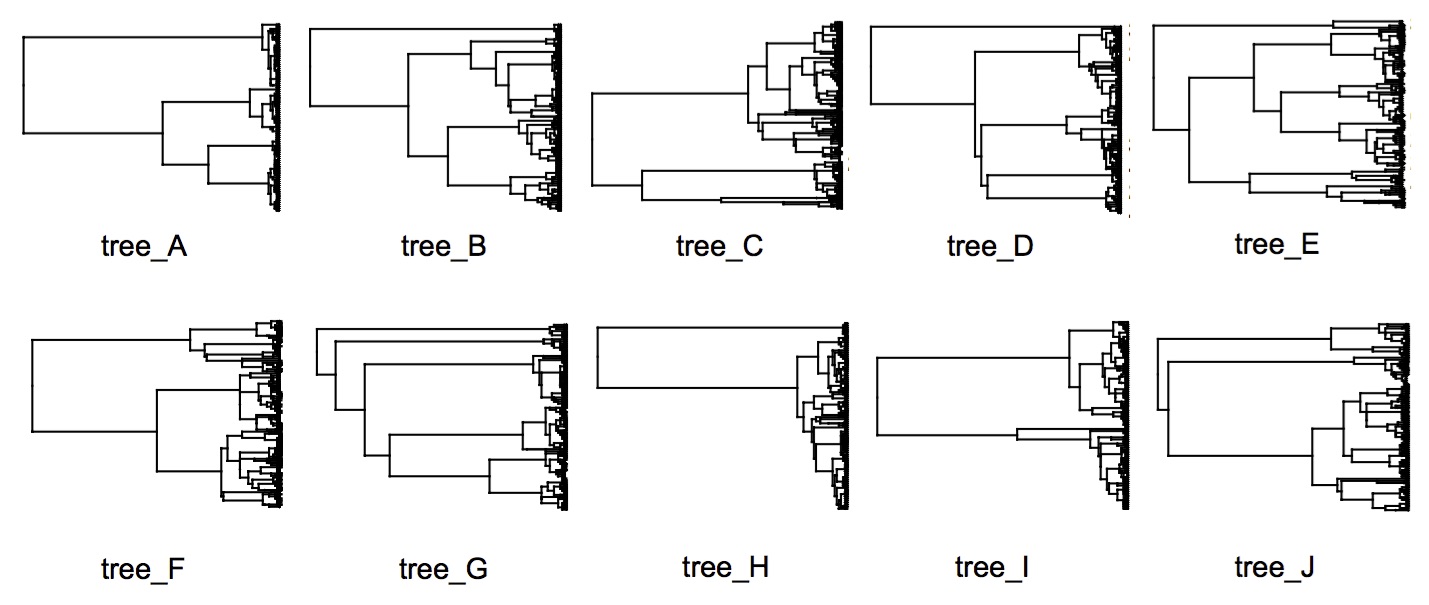

Supplement: Supplementary file 1 — Figure S1. Ten genealogies shown by rectangular phylogram. Since the branch lengths were rescaled while sequences being simulated, scale bars are not shown here. [file ECE3-5-5869-s001.jpg]

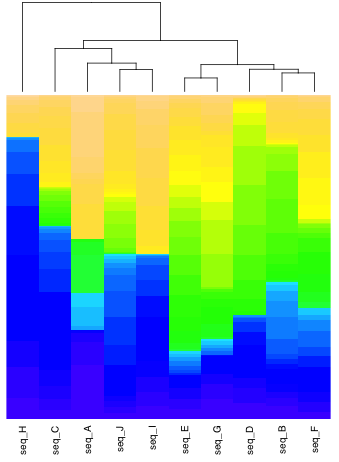

Supplement: Supplementary file 2 — Figure S2. Heatmap showing pairwise distances of the ten datasets (from seq_A to seq_J) together with hierarchical clustering. [file ECE3-5-5869-s002.tif]
