# Supplementary material for: A simulation study of sample size for DNA barcoding
Source: Ecol Evol. 2015 Dec 1;5(24):5869–79. doi: 10.1002/ece3.1846 (PMC4717336; doi:10.1002/ece3.1846)
Supplement: Supplementary file 4 — Data S2. Histograms showing distributions of nucleotide diversity values from all the datasets except seq_J. [file ECE3-5-5869-s004.docx]

seq_A:

seq_B:

seq_C:

seq_D:

seq_E:

seq_F:

seq_G:

seq_H:

seq_I:

seq_K:

seq_L:
