# Supplementary material for: A simulation study of sample size for DNA barcoding
Source: Ecol Evol. 2015 Dec 1;5(24):5869–79. doi: 10.1002/ece3.1846 (PMC4717336; doi:10.1002/ece3.1846)
Supplement: Supplementary file 5 — Data S3. Boxplots showing the numbers of haplotypes from all the datasets except seq_C. [file ECE3-5-5869-s005.docx]

seq_A:

seq_B:

seq_D:

seq_E:

seq_F:

seq_G:

seq_H:

seq_I:

seq_J:

seq_K:

seq_L:
